# Threats to epistemic agency in young people with unusual experiences and beliefs

**DOI:** 10.1007/s11229-021-03133-4

**Published:** 2021-03-30

**Authors:** Joseph W. Houlders, Lisa Bortolotti, Matthew R. Broome

**Affiliations:** 1 Department of Philosophy, University of Birmingham, 32 Pritchatts Road, Edgbaston, Birmingham, B15 2TT United Kingdom; 2 Institute for Mental Health, University of Birmingham, 52 Pritchatts Road, Edgbaston, Birmingham, B15 2TT United Kingdom

**Keywords:** Youth mental health, Epistemic injustice, Sense of agency, Therapeutic relationship, Autobiographical narrative

## Abstract

A good therapeutic relationship in mental health services is a predictor of positive clinical outcomes for people who seek help for distressing experiences, such as voice hearing and paranoia. One factor that may affect the quality of the therapeutic relationship and raises further ethical issues is the impact of the clinical encounter on users’ sense of self, and in particular on their sense of agency. In the paper, we discuss some of the reasons why the sense of epistemic agency may be especially fragile in young people with unusual experiences and beliefs. We argue that it is important to identify and avoid behaviours that can undermine young people’s contributions as epistemic agents in the clinical encounter.

## Introduction

Young people are increasingly struggling with mental health problems, and they are finding it hard to access the help they need (Erskine et al., 2017). When they get professional help, they are offered treatment and support. The quality of the therapeutic relationship that develops during the encounter is particularly important for those with unusual experiences and beliefs, such as hearing voices and paranoia, because positive engagement with services in general and with individual clinicians in particular is a predictor of good clinical outcome (e.g., Birkhäuer et al., 2017; Corsico et al., 2017; Farrelly & Lester, 2014; Laugharne et al., 2011). In the clinical encounter, the young person needs to feel listened to and understood and to develop a trusting relationship with the clinician. In this paper, we discuss the potential for the clinical encounter to affect young people’s sense of self and in particular their sense of agency, and we suggest that, in this context, undermining epistemic agency (i.e., the capacity to produce and share knowledge competently and authoritatively) is a form of injustice that may compromise both the users’ trust in services and the quality of the therapeutic relationship between young people and medical professionals.

Young people with unusual experiences and beliefs deserve special consideration because their epistemic agency is under multiple threats. First, the identity of young people in general is still fluid and more likely to be shaped by social interactions with power imbalances—the effects of challenging the sense of agency of a young person may be greater and more pervasive than the effects of challenging the sense of agency of a person whose identity is more securely established (Blakemore, 2019). Second, the sense of agency of people with unusual experiences and beliefs—who are considered as neurodivergent—may be more precarious due to the fact that unusual experiences and beliefs often undermine the sense of oneself as someone capable, efficacious, and in control; thus, young people with such experiences and beliefs may be more inclined to question their own thoughts.

Let us introduce the form of injustice that interests us here, inspired by the work of Miranda Fricker (2009):

*A* is wronged when:

1. It is believed by *B* that *A* is unable to exercise epistemic agency;
2. *B*’s belief is due to one aspect of *A*’s identity triggering a social prejudice against people with that identity being able to exercise epistemic agency;
3. *B*’s belief that *A* is unable to exercise epistemic agency negatively affects *A*’s ability to exercise epistemic agency and undermine *A*’s epistemic status.

This form of epistemic injustice may have more harmful consequences when there is an imbalance in the power relation between *B* and *A* which favours *B*, as is the case of a clinical encounter.

Let us start with a non-clinical example of epistemic injustice. Carla is a politician. During discussions with her allies and opponents on a heated policy issue, her opinion is dismissed. This is due to the other discussants harbouring a prejudiced view of women in politics—e.g., that women cannot be knowledgeable and authoritative in matters of policy. As a result of being routinely interrupted and dismissed, Carla comes to doubt her own worth and stops expressing her views. Carla’s gender is the aspect of her identity that triggers social prejudice against her agency, but we can imagine that other aspects of Carla’s identity, such as being of a certain age, belonging to an ethnic minority, being homosexual, or being overweight, could also contribute to the injustice she experiences. In the example, it is Carla’s epistemic agency that is compromised, in particular, her capacity to share knowledge competently and authoritatively, but other aspects of her agency could also come into question.

What do we mean by the sense of agency? The sense of agency broadly construed is the sense that one can intervene in one’s physical and social environment and shape one’s life to some extent. There are different dimensions to agency: we attribute *epistemic* agency to a person when we believe the person has the capacity to produce and share knowledge competently and authoritatively, and we attribute *moral* agency when we believe that the person has the capacity to act and make choices autonomously, regarding the person at least in part responsible for those actions and choices. The moral dimension of agency attributions can also give rise to injustices, but here we shall focus on the epistemic dimension.

What forms of injustice can people experience when they are not attributed agency at all or their sense of agency is undermined? The injustice experienced when epistemic agency is challenged occurs when factors that should not be directly relevant to a person’s testimony being assessed as credible and authoritative contribute to challenging or dismissing that person’s testimony (“Women do not understand politics”, “Young people are inexperienced”, “People who are overweight lack self-control”, etc.). Factors include, but are not exhausted by, gender, sexual orientation, socioeconomical status, ethnic origin, physical appearance.

Traditionally, young age and poor mental health have been considered as legitimate reasons to undermine people’s credibility and responsibility because they have been linked to absences or failures of rationality and autonomy, which explains the strong association between mental illness and insanity in some of the psychiatric and philosophical literature—an association often challenged in recent work (see e.g., Bortolotti, 2013). Indeed, in paradigmatic cases of gaslighting (Spear, 2019), attributing mental illness or mental instability to a person is a means of discrediting their testimony and creates an opportunity to take advantage of them (as in George Cukor’s 1944 film, *Gaslight*). However, the assumption that young people and people with unusual beliefs and experiences are irrational and thus lack the capacity to produce and share knowledge competently and authoritatively is often mistaken. Rationality comes in degrees and local failures of rationality—which are confined neither to young people nor to people who have unusual experiences and beliefs—may not have implications for overall judgements of rationality (see e.g., Bortolotti et al., 2012). This suggests that undermining the sense of epistemic agency of young people with unusual experiences and beliefs can qualify as a form of injustice.

## Why young people?

Our focus on youth is deliberate and stems from a variety of interrelated considerations. A basic one is that most mental health issues that are seriously debilitating and severely affect quality of life start manifesting themselves at a young age and this is especially true of the type of mental health issues we have chosen to focus on, which manifest in unusual experiences and beliefs (e.g., Marwaha et al., 2016). More relevant to our specific interest in the therapeutic relationship developing between clinicians and users of mental health services, youth is one of several factors that might affect, often negatively, the attitude of the clinician towards users and contribute to users developing a negative sense of themselves or questioning their sense of agency. This might result in young people coming to believe that they lack the capacity to intervene in the world and pursue their goals, something that negatively affects clinical outcomes as well. We know that shared decision-making and empowerment are linked to good therapeutic relationships and clinical outcomes, and hence, if young people are unable to contribute to the decision-making process because they feel powerless, then the quality of their therapeutic relationships with medical professionals and their clinical outcomes may be compromised. In addition, a sense of agency is arguably required for the success of certain treatments–for example, cognitive behavioural therapy and family therapy. Alongside the clinical argument, there is a powerful ethical argument for preserving the young person’s sense of agency: ideally, care plans should be co-produced by clinicians and service users, but co-production will remain an ideal if the young person has no sense that they have the capacity to contribute to a discussion about their treatment and is not given an opportunity to make such a contribution.

In the mental health context, the manifestation of behaviours such as hearing voices or expressing paranoid beliefs—behaviours that are often regarded as symptomatic of a mental disorder—can also be factors causing epistemically unjust attitudes and leading to a person being silenced, dismissed, or denied agency. Without justification, perceived irrationality in one area of mental life (“This person cannot distinguish between imagination and reality”) is generalised to other areas of decision making and agency that may actually be unaffected by the person’s poor mental health (Bortolotti et al., 2012). Challenging agency in people with unusual experiences and beliefs may be even more pronounced if people are young. This is due to the common association in popular culture between youth and negative features such as restlessness, unreliability, inconsistent behaviour, attention-seeking behaviour, laziness, recklessness, lying. An example would be the press’s use of the term “snowflakes” to refer to young people (e.g., Knapton, 2018), or the stereotype that young mothers are irresponsible and unable to take good care of themselves and their children (e.g., Ellis-Sloan, 2014).

Two further issues make youth an ideal focus for an analysis of epistemic injustice. First, identity. People’s attitude towards young people affects the way young people see themselves, what we may call their “self-image” or “self-conception”. Being constantly dismissed may lead people to revise their conception of themselves, either at a conscious or unconscious level, negatively affecting their self-esteem and causing them to come to believe that they have nothing to contribute or that their experiences, feelings, or beliefs are not to be trusted. The dangers of self-bias and self-stigma are likely to be heightened among young people who may not have yet a stable and fully developed identity and whose self-conceptions may be more likely to be influenced by environmental and social factors, and by the attitude of adults perceived as having relevant expertise in some domain, such as parents, teachers, and doctors. Some psychologists argue that a person’s identity is shaped by beliefs and experiences that they acquire in young adulthood (e.g., Addis & Tippett, 2008), precisely the period of life we are considering, and thus threats to young people’s sense of agency can have wide ranging consequences.

## The sense of self or identity

What constitutes a sense of self? The most persuasive answers to this question embrace the complexity of the phenomenon, i.e. acknowledging that many disparate elements may coalesce into what is felt to be one’s sense of self. These constituting elements will modulate, e.g. diminish, augment or alter qualitatively, other constituting elements.^1^ The following is a (non-exhaustive) list of proposed elements of a sense of self: memory; bodily awareness; interpersonal relationships; cultural norms; affect; and conscious self-assessment. Autobiographical narrative and perceived agency (inclusive of epistemic agency) are also important aspects of a sense of self.

In some influential contributions to the psychological and philosophical literature on memory and the self (Goldie 2012; Hydén and Örulv 2009; Schechtman 1996, 2007), ‘sense of self’ or ‘identity’ refers to beliefs about oneself answering two basic questions: (a) *Which* person am I? and (b) *What type of* person am I? Thus, the sense of self comprises both beliefs about one’s life story (e.g., when one was born and who one’s parents and siblings are) and beliefs about one’s personality traits and psychological dispositions (e.g., whether one has a generous disposition and is good at playing football). Sense of self is defined on the basis of *continuity*, that is, seeing oneself as extended in the past and the future; and *distinctiveness*, that is, seeing oneself as different from others. In both continuity and distinctiveness, autobiographical narratives play a pivotal role (Bortolotti & Sullivan-Bissett, 2018).

There are multiple ways in which a sense of self can be created, diminished, augmented, and perhaps even lost: for example, through interpersonal relationships, for instance with primary caregivers, friends, colleagues; and through pivotal life events, relocations, employment, trauma, and so on. Here we consider the relationship between the sense of self (especially the sense of epistemic agency) and autobiographical narratives*.* The focus on autobiographical narratives should not be taken to suggest that they are viewed as more important in creating or maintaining of a sense of self than the other aspects of a sense of self suggested above. It is, however, informed by a substantial interdisciplinary consensus between psychology (e.g., Bruner, 1991), philosophy (e.g., Dennett 1992), and psychodynamic therapy (e.g., Schafer, 1989) that autobiographical narratives are linked to feelings of selfhood. This is partly because the autobiographical narrative brings to the fore the continuity and distinctiveness of the self.

The sense of self is subject to subtle alterations, as well as to more extreme ones, depending on personal, circumstantial, and environmental factors. It appears to be prone to a greater degree and frequency of alterations earlier on in life. For example, interactions with family members and peers are likely to have a profound effect on a young person’s sense of self. By the same token, we expect there to be greater fixity in one’s sense of self as one grows older. That being said, independent of age, there are those for whom the sense of self is relatively fixed, with only minor variations occurring over time; and there are those for whom regular radical changes in sense of self are to be expected. This discussion builds to a consideration of how, in the clinical encounter, the young person’s autobiographical narrative may be shaped, at a time when their sense of self is still developing. An existing autobiographical narrative is likely to be challenged by mental health struggles and by figures of authority calling into question aspects of the person’s reports and experiences.

## Narratives and the sense of self

Let us move to self-narration, and how it relates to the sense of self. Stated simply, self-narration is the process of telling stories about one’s own life. Shaun Gallagher’s definition of narrative is useful in the context of an analysis of the clinical encounter:

I will take narrative in a wide sense, to include oral and written communications and self-reports on experience. In this regard narrative is more basic than story, and not necessarily characterized by the formal plot structure of a story. (Gallagher, 2007, p. 203).

This understanding of narrative suggests that most of us are in a near-constant state of self-narration. For example, when we come home and talk about our days (“You’ll never guess what happened to me on the train home”), or when we remind ourselves what we have been doing and are intending to do (“Lately I have been distracted and in a bad mood, but this week I am going to take steps to improve this”). To restrict the discussion to the form of self-narration which is likely to affect the sense of self, we shall confine our attention to *meaningful* self-narration—that is, stories that one tells about oneself which *one feels to be significant*, i.e. to be defining, or prototypical. The feelings that are connected to these stories may be more strongly felt—resulting in the sense that the stories themselves are important. So, meaningful self-narration is a sub-type of self-narration in virtue of how it is experienced by the person who tells it. Self-narration which is felt to be significant may more plausibly affect a sense of self, in negative and positive ways, because it is felt or reasoned to be more salient. In experiencing the story as salient, there is reason to think that it will be attended to more regularly, e.g., brought up in conversation and reflected upon more often.^2^

Consider the following examples of meaningful narratives. People may have narratives about themselves which cast them as “a fighter”, as “unlucky”, as “a coward”, as “a winner”, as “uninteresting”, and so on. These overarching summaries of oneself may be evidenced with certain exemplary stories. So, in the case of the fighter, a person may refer to times when they have had to work very hard to bring about the outcome they wanted, such as protesting the closure of a local library to the point of being arrested. Some people will have very specific, meaningful self-narratives which they view as defining. For example, a person who moved continents, and worked minimum-wage jobs to support their family, may understand this specific story as defining of their character as a whole.

## Narrative co-construction and the sense of agency

There are two important points concerning meaningful self-narration and its relation to a sense of self. First, the stories one tells about oneself are not insulated from the stories that other people tell—far from it. Second, the sense of agency is important for the formation of an autobiographical narrative (“Am I the hero of this story?”) and, at the same time, may be constrained or enhanced by the autobiographical narrative.

To elaborate on the first point, who is the author of the self-narrative? Gallagher summarises Ricoeur’s views on how autobiographical narratives are interlinked:

[Ricoeur] emphasizes the fact that one's own self-narrative is always entangled in the narratives of others, and that out of this entanglement comes a unified life narrative that helps to shape the individual's continuing behaviour. (Gallagher, 2007, p.212)

These “others”, with whom our own narrative is “entangled”, may be family, friends, and perhaps trusted professionals, including clinicians. Stories that other people tell about *themselves* may inform our own story. For example, if they are a relative and they self-narrate in such a way as to lead us to think that they are happy, this may impact our own self-narration, e.g. we may begin to see ourselves as conducive to, or at least not disruptive of, their happiness. Also, ‘others’ may also tell stories about *us*, thus contributing to our self-narration (providing we incorporate their stories in one way or another in our story). So, suppose that our parents comment that we were always badly behaved, and interpret our current behaviour in light of this. Our parents’ interpretation of our behaviour may well impact the story that we tell about ourselves. Of course, it will not necessarily lead us to think of ourselves as rebellious or anti-authoritarian; it could be that our parents offering this interpretation leads us to feel more strongly that the opposite is true. That being said, the interpretations of others may still be constituting of our sense of self, even if they are not believed.

How do autobiographical narratives impact on the sense of agency? It has been suggested that certain types of autobiographical narrative may change both the amount and kind of possibilities for action that one experiences (e.g., Miller Tate, 2019; Ratcliffe, 2016; Ratcliffe & Broome, 2012). A sense of agency is inextricably linked to one’s sense of *what actions are possible*: to feel that you can carry out an action requires the feeling that the action is possible. Here ‘action’ includes not just overt physical behaviour, for example frying an egg or climbing a hill, but also the performance of certain mental acts, for example making judgments and predicting future events.

Experiences may issue implicit invitations for actions. For example, one’s experience of a hill may contain an implicit invitation to climb it. So, when we see a hill our experience includes more than just visual data: it also includes the possibilities for action that are afforded by the hill. It may be felt to offer numerous possibilities all at once, e.g. for climbing, rolling down, getting a good view from, etc. The possibilities are not necessarily consciously attended to (they are not, for example, listed all in the person’s head), but nevertheless it is suggested that they implicitly form part of the experience. The types of possibilities and the extent to which they are felt will, of course, vary from person to person. For example, if one is in the process of building a new railway, the possibility of tunnelling through the hill may form part of the experience. If one has a broken leg the kinds of possibilities the hill affords may be decreased. And, as a final example, if one is suffering from symptoms of depression, the experience of a hill may lack possibilities for any positive, enjoyable activity.

Autobiographical narratives may increase, decrease, or bring qualitative change to, one’s sense of possibilities for action, as they construct a version of oneself which implies, or explicitly states, that certain kinds of action are possible, and that other kinds are not. For example, if your autobiographical narrative casts you as an adventurous person, certain kinds of possibilities may be present in your experience. These are unlikely to be present for those whose autobiographical narratives cast them as being very cautious and risk averse. There are different ways of suggesting how autobiographical narrative may have this effect. One plausible way is via emotions or existential feelings (for an example of the latter, see Ratcliffe, 2016). For example, when one feels generally happy, one is more likely to experience one’s surroundings as offering possibilities for pleasant, fun, activities: e.g., the park is experienced as a place for lounging around eating ice cream, rather than a place where you might be mugged.

What does this mean for *epistemic* agency? The relationship between autobiographical narrative and agency becomes more complicated when we start to think about felt possibilities for being competent and authoritative in producing and sharing knowledge. For instance, an autobiographical narrative could cast one as incapable of distinguishing the real from the imagined because one hears voices and has other unusual experiences. Such a narrative is likely to bring about fearfulness and self-doubt. Alternatively, an autobiographical narrative could cast one as someone who has struggled in the past to discern what is real, but who has undertaken work to address this. Such a narrative may inspire feelings of hope. These affective responses may alter one’s general sense of possibility for exercising epistemic agency. It is plausible, also, that these alterations then feed back into the autobiographical narrative, generating a looping effect (Ratcliffe, 2016).

## Back to epistemic injustice

So far, we have defined the sense of self and suggested that meaningful autobiographical narratives, which are often co-constructed, may alter the sense of agency intended as the subjective feeling of being able to carry out what one intends to. For example, an autobiographical narrative may augment or diminish an existing sense of agency, or even cause it to change qualitatively.

It is now time to go back to the notion of epistemic injustice. In what circumstances are people at the receiving end of unjust treatment if their sense of agency is challenged? As we saw, self-narration has been strongly linked with a general sense of agency, which is plausibly included in one’s sense of self. Here, we are interested in how the story that one tells about oneself impacts the sense of oneself as an epistemic agent. The sense of agency (or perceived agency) has been defined in different ways in the psychological literature, where some uses of the phrase are broader, referring to the general tendency to initiate or have control over one’s actions, whilst other uses are more specific, referring to the sense that people initiate or are in control of a particular action. One basic description of the sense of agency is “the experience of controlling one’s own actions, and, through them, events in the outside world” (Haggard and Chambon 2012). People have a sense of agency if they see themselves as the initiators of their actions; they feel in control of their bodies and minds; and they feel they can intervene on their environment (Christensen et al. 2019; Tapal et al. 2017). With respect to thoughts and other mental states, the sense of authorship can also be added to the sense of agency, where people feel that thoughts do not just happen to be “in their heads”, but are also thoughts that they feel they can commit to and give reasons for (Bortolotti & Broome, 2009).

Objective agency is when an agent performs an action, whereas the sense of agency is when the agent feels that they are performing or have performed an action. We find in the literature some fascinating examples of cases in which agents feel in control of some external events, but did not actively intervene on them: people playing dice tend to believe that they can determine what number will come up if they are the ones throwing the dice; people practising voodoo believe that inflicting a pin on a doll will cause pain to some other person in the real world; and pedestrians tend to think that when they press the button at the crossing they cause the green light to appear, although most traffic lights are timed (Moore 2016). Some behaviours that are considered symptoms of mental disorders, such as passivity and thought insertion, are also often mentioned as examples of failure of the sense of agency. These are cases where the person is the initiator of a particular action or the source of a particular thought but do not feel they are and thus attribute the action or the thought to a third party (see for instance Gallagher, 2015; Bortolotti & Broome, 2009; Humpton and Broome 2016).

How do we *measure* the sense of agency? There are different measures depending on the type of feeling that is studied and the context in which it is studied. Some implicit measures (such as time perception) are more suitable to track the sense of agency that applies to specific actions. Focusing on the explicit measures which are based on people’s reports and can be biased, someone with a strong sense of agency would tend to agree with statements such as “I am in full control of what I do” or “I am completely responsible for everything that results from my actions” and disagree with statements such as “The outcomes of my actions generally surprise me” or “I am just an instrument in the hands of somebody or something else”. Perceived agency in its general sense is related to other constructs such as self-efficacy—described as “a generalized, positive belief in personal competence and ability to organize and execute desired behaviour” (Tapal et al. 2017)—but whereas self-efficacy is manifest in the sense that people can attain their goals, perceived agency is conceptually prior to that as it focuses on the capacity people have to initiate action and control their bodies, their minds, and the surrounding environment to some degree.

Although perceived agency does not coincide with objective agency and can be illusory, perceived agency is important in its own right because the feeling that one is performing an action, and is in control, or has performed an action in the past, and has thus been in control, can impact one’s future capacity to exercise objective agency, as we suggested at the end of the previous section. For instance, in a recent study on how biological models of depression affect people’s sense of agency it was found that “when people are pessimistic about their own prospects of overcoming a disorder or benefitting from treatment—which may be especially likely if they attribute their symptoms to biological causes—their negative outlooks can become self-fulfilling prophecies” (Lebowitz and Ahn 2017, p. 125). The study shows that, when an intervention is developed to educate users about the malleability of biological factors responsible for depression, users’ confidence in their chances to recover from depression increases, thanks to their sense of agency being enhanced. This has positive implications for clinical outcomes. In general, when people have a strong sense of agency, control, and self-efficacy, they enjoy better mental health, avoid experiencing feelings of helplessness after setbacks, and act more *like agents*, preserving their motivation to pursue their goals in adverse circumstances (Bandura, 1989). As a consequence, people are also more likely to perform satisfactorily and fulfil their goals. They tend to be more productive, more resilient, better at planning, and more effective at problem-solving (e.g., Alicke & Sedikides, 2009; Bortolotti et al., 2019).

As anticipated, epistemic agency is concerned with a person’s capacity to produce and share knowledge. People have a sense of epistemic agency if they feel that they enjoy competence, authority and credibility as knowers of their own experiences and feel that they master the conceptual resources necessary to understand their situation. In the philosophical literature (Fricker, 2009), the epistemic domain is one where distinct forms of injustice can occur, where people’s testimony is discredited for non-epistemic reasons (e.g. in *testimonial injustice*, one’s knowledge may be discounted due to gender or ethnicity) and where, due to non-epistemic factors, people lack the conceptual resources to interpret their own situation (e.g. in *hermeneutical injustice,* one may be excluded from a technical discussion regarding medication).

Recently, there has been some interest in applying epistemic injustice to the medical and especially the mental health context (e.g., Byrne, 2020; Crichton et al., 2017; Dotson, 2012). Our interest is in understanding the relationship between epistemic agency and epistemic injustice in the experiences of a group of people who may have their views discounted due to multiple reasons including illness, youth, ethnicity, poverty, and gender. How can social interactions, and more specifically interactions during clinical encounters, support or undermine a person’s sense of agency? The attitude people take towards each other has wide-ranging effects on their experiences of themselves and the world.

By our very attitude to one another we help to shape one another’s world. By our attitude to the other person we help to determine the scope and hue of his or her world; we make it large or small, bright or drab, rich or dull, threatening or secure. We help to shape his or her world not by theories and views but by our very attitude toward him or her (Løgstrup, 1997, p. 18)

The general approach a clinician takes to the young person they are working with (their ‘attitude’) can affect young people’s experience of themselves and their surroundings. Conversation analysis has already been applied successfully to interactions between clinicians and people experiencing psychotic symptoms. With conversation analysis we can explore the epistemic dimension of the clinical encounter: what is known, how it is known, who has the right to know it. The method consists in studying verbal and non-verbal communication, and reflecting on factors such as interruptions, tone, emphasis, and gaze, as well as on the content of the exchange (McCabe et al., 2002).

Some of the evidence on clinical encounters gathered by using conversation analysis offers a good insight into exchanges that have the potential to adversely affect people’s sense of agency. In exchanges between a psychiatrist and users with psychotic symptoms, for instance, users actively describe their experiences but often the doctor provides no response and avoids direct questions that users might have about their experiences. This suggests to the users—and to a neutral observer—that there is no engagement: the psychiatrist does not explain or discuss the relevant experiences despite these being important to the users and users seeking to know more about them.

[Patients with psychotic symptoms] clearly attempted to discuss their psychotic symptoms and actively sought information during the consultation about the nature of these experiences and their illness. When patients attempted to present their psychotic symptoms as a topic of conversation, the doctors hesitated and avoided answering the patients' questions, indicating reluctance to engage with these concerns. (McCabe et al. 2002, p. 1150)

As McCabe and colleagues notice, in the course of the conversation with the psychiatrist when a carer is also present, and users express concern about their experiences or ask for explanations (such as “why don’t people believe me when I say I am God?”), the psychiatrist often responds with hesitation (“hmm”), with another question (“what should I say now?”) or with a smile or laughter. This makes users feel that they are not taken seriously, and their concerns are not addressed.

Such exchanges in the clinical encounter point to the risk of testimonial injustice: the doctor fails to engage with the users’ account of their own experiences. Engagement does not require or imply an endorsement of the unusual experiences or of the unusual beliefs, but simply an acknowledgement that users share information about their experiences that is important to them and that their contributions and concerns matter to the success of the encounter.

There is also potential evidence of hermeneutical injustice because users are denied the conceptual resources to understand their own experiences—they ask questions that the doctor does not answer. As we saw, McCabe and colleagues notice that, in this form of exchange, although users actively attempt to talk about the content of their symptoms and ask about them, their questions are avoided. One effect of the psychiatrist not commenting or not answering the questions is that users do not feel that their reports are valued as meaningful contributions to the conversation. Another effect is that a different understanding of their experiences that could be encouraged by the psychiatrist is not made available to them.

People are usually taken to be credible and authoritative when they report the content of their own experiences and beliefs, that is, people are expected to know what their experiences and beliefs are and to report them accurately unless they have a reason to deceive. That does not mean that those experiences and beliefs can always be understood or shared by others, but that people are typically considered to be in a better position than anybody else to describe such experiences and beliefs. However, when people report *unusual* experiences and beliefs, the credibility and authority of their reports are no longer taken for granted due to the possibility of a substantial gap between how people take things to be and how things really are.

It is primarily because they report unusual experiences and beliefs that the epistemic agency of users with psychotic symptoms is questioned. But is questioning epistemic agency justified or is it an instance of epistemic injustice? That will depend on what triggers the challenge or the lack of response: if the attitude of the psychiatrist is determined by the person’s identity as a user of services with psychotic symptoms, then there does not seem to be a legitimate reason for epistemic agency to be undermined overall, because we should not assume that the epistemic agency of people with unusual experiences and beliefs is compromised by default. There are very good reasons to reject the assumption that rationality is globally affected by the presence of psychotic symptoms, and indeed we should treat cases in which reports of people’s experiences are not engaged with, but challenged or dismissed, as genuine cases, we would venture to say *textbook cases*, of epistemic injustice.

In their paper on epistemic injustice and the assessment of delusions, Sanati and Kyratsous (2015) present two cases of people who were experiencing distress and reported beliefs that were judged as delusional by their clinical team. Such beliefs turned out to be true and not to related to their delusions.

We think that the patients are not given the same credibility as a non-patient on the basis of having an illness that is so often associated with attributions of *irrationality*, *bizarreness* and *incomprehensibility*. The type of prejudice that these people are shown to be suffering is related with their social identity and has been persistently and diachronically featuring in social representations of schizophrenia and conceptions of delusions. It is also relevant that the lack of an integrative clinical model of understanding delusions, which does not focus so much on the experience of the person, is one main factor that makes these prejudices salient, interfering in the assessment of such mental states. (Sanati and Kyratsous, p. 483).

Further, in recent inquiries into the potential protective role of some delusional beliefs (e.g., Catone et al. 2015, 2017; Gunn & Bortolotti, 2018; Gunn & Larkin, 2020; Moffa et al. 2017), it is found that the delusion is not an incomprehensible speech act and can make reference to actual life events. It can arise after trauma or an otherwise significant event that has emotionally affected the person, such as abuse or bullying, and reflects the need for the person to respond to such disruptive experiences—it is meaningful and ‘makes narrative sense’.

[…] the delusion is not just a glitch, but its content relates powerfully to significant events in the person’s life and, at the time when it is first adopted, relieves the person of some heavy psychological burden. In particular, in at least some cases, delusion formation can be seen as a short-term protective response to disruptive and traumatising life events. (Gunn and Bortolotti 2018, p. 830).

Such contributions to the literature on delusions suggest that challenging or dismissing users’ reports of their own experiences without engagement is both an instance of epistemic injustice, and a missed opportunity to understand at least in part what they are going through and how best they can be helped. The idea that mental illness and in particular psychotic symptoms are evidence of global irrationality is culturally well-entrenched but very poorly supported by the evidence available to us. More to the point, given our focus here, challenging or dismissing users’ reports without engagement has significant risks for their sense of agency at a time when their sense of agency is already potentially undermined by their mental health struggles.

There are factors specific to young adults which make them especially susceptible to epistemic injustice during the clinical encounter. First, the clinician may have an unconscious bias related to youth, assuming that young people lack clear insight, or that what motivates them is not health-promoting, as when they are thought to prioritise respect from peers to good psychotherapeutic outcomes. Second, there are some harmful societal narratives about young people. As was flagged earlier, there are narratives about young people which—if unchallenged—could lead to see them as being overly sensitive, not knowing what they want, or being unwilling to work hard to achieve what they want. Third, as the young person’s sense of self is still forming, it is more susceptible to external assessment, in that it is more readily shaped by the opinions of others who are perceived as experts in some domain. To state this in terms of the narrative conception of the self, the interaction with the clinician may lead young people to alter the story that they tell about themselves for the worse, inferring from unsuccessful clinical encounters that their contributions are worthless and not worth engaging with. As was discussed earlier, a self-narrative can sensitize people to certain kinds of possibilities. The environment surrounding many clinical encounters is experienced in such a way as to consolidate a particular perception of the self, where one’s experience is not important, should not be trusted, and can be dismissed without significant loss.

## Conclusions and implications

The clinical encounter is the place where positive engagement can lead to good outcomes and is also the place where the young person’s agency may be enhanced or undermined, often through co-constructed self-narratives. When young people’s sense of epistemic agency is compromised in the context of the clinical encounter, this can have pervasive and durable effects on the quality and effectiveness of the therapeutic relationship and on the young people’s capacity to exercise their epistemic agency.

In this paper, we have made a case for the view that young people with unusual experiences and beliefs are potentially at a higher risk of harm from epistemic injustice than either young people with no such unusual beliefs and experiences or people with unusual beliefs and experiences in different age groups. This is due to the stigma associated with certain behaviours that are regarded as symptomatic of poor mental health, and societal prejudices towards youth. This is also due to the fact that young people are still developing their own identity and are thus more vulnerable to the effects of other people’s attitudes towards themselves.

Our suggestion is that empirical evidence about encounters between clinicians and young people with unusual experiences and beliefs should be gathered and analysed on the background of the risks we have identified. It is hoped that such information can provide the basis for training clinicians in leading mutually satisfying conversations with users, where the users’ interests and needs are acknowledged, and the users’ status as epistemic agents is not implicitly or explicitly undermined. We believe this may increase mutual trust as well as protect users’ sense of self. In particular, it is important to recognise the pivotal role that clinicians (alongside primary carers, teachers, and peer groups) have in shaping the autobiographical narratives of young people with unusual experiences and beliefs whose sense of agency is already under threat.

## Footnotes

1 A good example of this interactivity can be found in what is known as ‘the pattern theory of self’ (e.g. Gallagher 2018).

2 Whilst this suggestion seems reasonable, it is important to also acknowledge the effects on a sense of self that mundane, everyday self-narration may have (where what is said is not imbued with significance by the self-narrator); the stories we *unthinkingly* tell about ourselves, which are ascribed no importance, in all probability do also affect our sense of self (say, for example, if we have an unconscious tendency to cast ourselves as the victim in the stories we tell, or to gloss over the positive aspects of a story).
